# Risk of eating disorders in a representative sample of Italian adolescents: prevalence and association with self-reported interpersonal factors

**DOI:** 10.1007/s40519-021-01214-4

**Published:** 2021-05-20

**Authors:** Giulio D’Anna, Marco Lazzeretti, Giovanni Castellini, Valdo Ricca, Emanuele Cassioli, Eleonora Rossi, Caterina Silvestri, Fabio Voller

**Affiliations:** 1 Psychiatry Unit, Department of Health Sciences, University of Florence, Azienda Ospedaliero-Universitaria Careggi, Largo Brambilla 3, 50134 Florence, Italy; 2 Agenzia Regionale Di Sanità Della Toscana, Florence, Italy

**Keywords:** Adolescence, SCOFF, Family relationships, Bullying victimization, Sexuality, Psychological distress

## Abstract

**Purpose:**

Adolescence represents a critical period for the onset of eating disorders (EDs). The present study aimed to provide the prevalence of individuals at risk for EDs psychopathology in a representative population of adolescents aged 14–19 and to characterize this population regarding interpersonal and psychological factors.

**Methods:**

The percentage of participants at risk for EDs in a representative high school population was assessed through the SCOFF screening questionnaire (cut-off score: 3) in the total sample (N: 6551) and in gender-based subgroups for different body mass index (BMI) categories. Odds ratios for being at risk of ED (SCOFF ≥ 3) were esteemed in a multivariable analysis including self-reported parental education, quality of family and peer relationships, bullying victimization, age at first sexual intercourse, and psychological distress.

**Results:**

A SCOFF score ≥ 3 was found in 31.0% of participants (boys: 19.4%; girls: 44.6%), with a greater prevalence among higher BMI categories. Bad family relationships, being bullied, having the first sexual intercourse before the age of 14, and experiencing high distress were associated with this risk condition. Among girls, bad peer relationships were associated with a low-risk SCOFF score.

**Conclusion:**

A remarkable percentage of adolescents reported significant body image or eating concerns. Screening programs are deeply needed, and particular attention should be devoted to interpersonal factors, such as the quality of family relationships and interactions with peers, which represent potential indicators of this vulnerability.

**Level of evidence:**

Level V – Cross-sectional study.

## Introduction

Recent studies seem to demonstrate an increased incidence of cases of eating disorders (EDs) among adolescents in the last decades [1]. However, a precise estimate of the prevalence of subthreshold and clinical conditions in this high-risk age interval is still under-addressed in European countries [2], and data from previous Italian studies may benefit from an update [3–6]. In fact, early detection of ED psychopathology is fundamental for a timely treatment and a reduction of chronicity [7]. For this reason, an accurate characterization of at-risk conditions—including different environmental, interpersonal, and psychological factors clustering with them—may contribute to a more precise estimation of the number of subclinical conditions or untreated subjects with EDs [2, 8].

On the one hand, the presence of shared risk factors between disordered eating and a condition of overweight among adolescents has been clearly outlined [9], and a history of overweight or obesity is potentially associated with the development of EDs [10], including dietary restraint and subsequent weight loss [11].

When exploring this framework, perceived social norms and family environment have shown to influence both eating behavior and body image concerns [12], and—since a cultural influence in EDs is clearly established [13]—socioeconomic determinants are probably involved in a complex and multifaceted manner. In this sense, parental education level has shown a clear gender- and-disorder-specific predictive value in a large naturalistic cohort, differently from indicators such as income or social class [14].

Regarding interpersonal and psychological factors, the development of ED symptoms may be related to difficulties in family interactions [15–17]– possibly involving the widely described role of dysfunctional attachment patterns [18]—but also to psychological distress [19], and relationships with peers [17, 20, 21]. More in details, the latter were found to influence weight-related behavior [20], and bullying victimization was associated with weight and body image concerns among adolescents [21]. In fact, premorbid social difficulties in EDs include submissiveness and proneness to dysfunctional social comparison [22]. Moreover, experiencing family and peer teasing in early adolescence is associated with the development of dysfunctional weight control tied to body image dissatisfaction [17]. To conclude, the quality of early sexual experiences has already been reported to be interconnected with EDs psychopathology [23, 24], early sexual activity being associated with bulimic-type pathology in middle adolescence [25]. Considering that ED patients are known to show abnormal emotional reactivity associated with body dissatisfaction in presence of social stressors [26], an early assessment of the abovementioned relational factors may enrich the epidemiological data available, coherently with the interpersonal model of EDs.

The present study aimed to produce the first evaluation of the prevalence of EDs symptoms in a representative population of Italian adolescents, and to explore differences in risk for ED psychopathology across known risk factors related to family environment, peer relationships, psychological distress, and body weight.

## Methods

The present investigation is part of the larger EDIT (Epidemiologia dei Determinanti dell’Infortunistica stradale Toscana) study on at-risk behavior among Italian adolescents.

In 2018, Agenzia Regionale di Sanità (ARS) Toscana collected data from 6824 individuals aged 14–19 years who were attending 85 public high schools of Tuscany (Italy). To avoid an inadequate representation of age, gender, type of school attended, and geographic region, institutions were selected through a randomization process based on socio-demographic data provided by the Regional Board of Studies. For each school, five classes were randomly chosen from different sections to avoid biases related to specific characteristics of each section.

The survey procedure was explained before inclusion in the study, and written informed consent was collected from participants, and from their parents or legal guardians (for individuals younger than 18 years). Enrolled subjects answered a self-reported questionnaire on an electronic form during school hours, and confidentiality was always ensured. The study protocol was approved by the Ethics Committee of the organizing institution.

### Measurements and variables

Enrolled individuals reported their age, gender, height, and weight. Body mass index (BMI) was obtained from self-reported weight and height. BMI normative percentiles outlined four categories within the total sample [27, 28]: “Underweight” (BMI < 5th percentile), “Normal weight” (5th–85th percentile), “Overweight” (85–95th percentile), and “Obesity” (> 95th percentile).

Participants were asked to fill the Italian version of the SCOFF questionnaire, a 5-item survey with dichotomous answers (“Yes”: 1 point; “No”: 0 points) for the screening of EDs [29, 30]. Questions refer to the acronym SCOFF and investigate feelings of sickness related to being uncomfortably full (Sick), perceived loss of control when eating (Control), a recent loss of at least six kilograms in a three-month period (One stone), perceiving oneself as fat (Fat), and the idea that food is dominating the respondent’s life (Food). Individual scores range from 0 to 5, with higher scores indicating a higher number of ED symptoms. The cut-off score for risk of an ED in the Italian version is 3, with a sensitivity of 97.0%, and a specificity of 87.5% in detecting EDs [30].

Family environment was evaluated through self-reported highest parental education level (“Graduated” or “Not graduated” from university), and through the perceived quality of family relationships, categorized as “Good” (if “Very good” or “Quite good”), “Intermediate” (if self-reported as “So and so”), or “Bad” (if “Not very good” or “Not good at all”).

Peer relationships were investigated through a self-reported questionnaire and categorized as described for family relationships. In addition, the survey asked whether participants had been physically or verbally bullied by peers, including cyberbullying, during the previous year (“Yes” or “No”), and the age at the time of the first complete sexual intercourse (pragmatically categorized as < 14 years or ≥ 14 years, which is the usual age at the beginning of high school, and, therefore, the minimum age in the analyzed sample).

Perceived psychological distress was evaluated through the K6 scale [31]. This screening scale contains six Likert-format items which assess feelings of hopelessness, depression, uselessness, nervousness, restlessness, and feeling overwhelmed in the past 30 days (from 1, “Never”, to 5, “Always”). Scores > 18 were classified as “High distress”. The K6 scale proved to have adequate psychometric properties in an adolescent population [32].

### Data analysis

Participants were excluded from analysis if they had not answered the SCOFF questionnaire. After excluding individuals who did not provide consent or did not answer the SCOFF questionnaire, the sample was checked for selective participation, to confirm that it did not significantly differ from the initial one regarding randomization criteria (age, gender, geographic region of origin, and type of school attended). Descriptive statistics—divided by gender—were presented as absolute and relative frequency. The primary outcome variable was the SCOFF score, with a score ≥ 3 indicating a risk condition for EDs. Chi-square tests (*χ*^2^) were used to assess the distribution of SCOFF scores above and under the cut-off of 3 within different BMI categories. For each gender, chi-square (*χ*^2^) tests evaluated differences between participants with or without a SCOFF score ≥ 3 regarding self-reported items, and a multivariable logistic regression was performed to outline factors predicting risk for EDs (SCOFF score ≥ 3), expressed as adjusted odds ratios (AORs) with 95% confidence intervals (CIs). The null hypothesis was rejected for alpha < 0.05. Analyses were performed using Stata 14 [33].

## Results

A total of 273 subjects did not fill the SCOFF screening scale, and they were, therefore, excluded from analyses. The final sample included 6551 individuals: 3540 boys (54.0%), and 3011 girls (46.0%).

Individuals with a SCOFF score ≥ 3 were 31.0% of the total sample (*n* = 2030): 19.4% of the boys (*n* = 687), and 44.6% of the girls (*n* = 1343). A SCOFF score of 5 was observed in 2.4% of the total sample (n = 154), in 1.2% of the boys (*n* = 41), and in 3.8% of the girls (*n*: 113). In the total sample, the relationship between BMI categories and SCOFF scores showed an increased prevalence of SCOFF scores ≥ 3 for higher BMI categories (*χ*^2^ = 33.90, *p* < 0.001; Table 1). Similar trends were detected for the subgroups of boys (*χ*^2^ = 80.37, *p* < 0.001; Table 2) and girls (*χ*^2^ = 18.30, *p* < 0.001; Table 3).

**Table 1:** Percentage of individuals under and above the SCOFF cut-off score for each body mass index category (total sample)

BMI category	SCOFF < 3	SCOFF ≥ 3
Underweight (*n* = 175)	134 (76.6%)	41 (23.4%)
Normal weight (*n* = 5180)	3635 (70.2%)	1545 (29.8%)
Overweight (*n* = 836)	537 (64.2%)	299 (35.8%)
Obesity (*n* = 153)	82 (53.6%)	71 (46.4%)
Not available (*n* = 207)	133 (64.3%)	74 (35.7%)
Total (*N* = 6551)	4521 (69.0%)	2030 (31.0%)

*BMI* body mass index

**Table 2:** Percentage of boys under and above the SCOFF cut-off score for each body mass index category

BMI category	SCOFF < 3	SCOFF ≥ 3	Individuals within BMI category
Underweight (*n* = 57)	50 (87.7%)	7 (12.3%)	1.6%
Normal weight (*n* = 2699)	2259 (83.7%)	440 (16.3%)	76.2%
Overweight (*n* = 562)	399 (71.0%)	163 (29.0%)	15.9%
Obesity (*n* = 99)	59 (59.6%)	40 (40.4%)	2.8%
Not available (*n* = 123)	86 (69.9%)	37 (30.1%)	3.5%
Total (*N* = 3540)	2,853 (80.6%)	687 (19.4%)	100%

*BMI* body mass index

**Table 3:** Percentage of girls under and above the SCOFF cut-off score for each body mass index category

	SCOFF < 3	SCOFF ≥ 3	Individuals within BMI category
Underweight (*n* = 118)	84 (71.2%)	34 (28.8%)	3.9%
Normal weight (*n* = 2481)	1376 (55.5%)	1105 (44.5%)	82.4%
Overweight (*n* = 274)	138 (50.4%)	136 (49.6%)	9.1%
Obesity (*n* = 54)	23 (42.6%)	31 (57.4%)	1.8%
Not available (*n* = 84)	47 (56.0%)	37 (44.0%)	2.8%
Total (*N* = 3011)	1668 (55.4%)	1343 (44.6%)	100%

*BMI* body mass index

In the total sample, 175 subjects had a condition of underweight (2.7%), 5,180 subjects had a normal weight (79.1%), 836 subjects were overweight (12.8%), and 152 (2.3%) had a condition of obesity. BMI was not available for 207 subjects (3.2%). The gender-specific distribution of BMI categories is shown in Tables 2, 3.

The gender-specific relationship between SCOFF scores and self-reported variables is reported in Table 4. In both genders, a different distribution was detected for each of the variables presented, except for parental education level. More in details, individuals with high SCOFF scores reported a lower quality of family relationships, more bullying victimization, a younger age at first sexual intercourse, and they referred to experience higher levels of psychological distress. The self-reported quality of peer relationships showed an asymmetrical distribution, with a lower prevalence of high SCOFF scores among those individuals reporting good peer involvement (Table 4).

**Table 4:** Characterization of relational and behavioral variables in individuals with and without a SCOFF score ≥ 3, divided by gender

	Boys				Girls			
	SCOFF < 3	SCOFF ≥ 3	χ^2^	AOR for SCOFF ≥ 3 and 95% CI (*n* = 3169)	SCOFF < 3	SCOFF ≥ 3	χ^2^	AOR for SCOFF ≥ 3 and 95% CI (*n* = 2775)
*At least one graduated parent*								
No	1813 (80.6%)	437 (19.4%)	0.01	1	1045 (54.4%)	875 (45.6%)	0.60	1
Yes	821 (80.6%)	197 (19.4%)		0.98 (0.81–1.20)	515 (56.0%)	405 (44.0%)		0.97 (0.82–1.15)
*Quality of family relationships*								
Good	2512 (81.7%)	564 (18.3%)	68.03***	1	1420 (58.4%)	1013 (41.6%)	81.14***	1
Intermediate	248 (76.5%)	76 (23.5%)		0.93 (0.68–1.27)	171 (43.6%)	221 (56.4%)		1.34 (1.06–1.70)*
Bad	65 (60.7%)	42 (39.3%)		1.90 (1.20–3.00)*	67 (39.6%)	102 (60.4%)		1.41 (1.00–2.01)*
*Quality of peer relationships*								
Good	2581 (81.2%)	596 (18.8%)	32.54**	1	1451 (56.4%)	1121 (43.6%)	47.63**	1
Intermediate	211 (73.5%)	76 (26.5%)		1.20 (0.87–1.64)	161 (46.8%)	183 (53.2%)		1.08 (0.84–1.39)
Bad	35 (74.5%)	12 (25.5%)		0.69 (0.33–1.49)	40 (53.3%)	35 (46.7%)		0.57 (0.35–0.95)*
*Bullied in the previous year*								
No	1897 (84.6%)	345 (15.4%)	11.12***	1	1062 (62.0%)	650 (38.0%)	11.45***	1
Yes	892 (73.0%)	330 (27.0%)		1.75 (1.45–2.11)*	567 (45.4%)	683 (54.6%)		1.67 (1.42–1.96)*
*Sexual debut before 14 years*								
No	2790 (81.2%)	645 (18.8%)	29.34***	1	1641 (55.9%)	1295 (44.1%)	11.71*	1
Yes	63 (60.0%)	42 (40.0%)		2.28 (1.47–3.54)*	27 (36.0%)	48 (64.0%)		1.88 (1.11–3.16)*
*Distress*								
Low	2574 (82.8%)	536 (17.2%)	77.23***	1	12.9%)	760 (37.1%)	143.69***	1
High	279 (64.9%)	151 (35.1%)		2.17 (1.69–2.79)*	382 (39.6%)	583 (60.4%)		2.21 (1.86–2.64)*

Adjusted odds ratios for having a SCOFF score ≥ 3 are presented (multivariable model)

*AOR* adjusted odds ratio, *CI* confidence interval *p < 0.05, **p < 0.01, ***p < 0.001. Percentages refer to the single answer for each gender (total number varies due to missing data)

Bold is used to highlight statistically significant results

In both subgroups, AORs for having a SCOFF ≥ 3 outlined a significant association with bad family relationships, being bullied, being younger than 14 years at first sexual intercourse, and experiencing high distress (Table 4). Gender-specific patterns were observed for girls with an intermediate quality of family relationships (a risk factor not observed for boys), and for girls reporting bad peer relationships—a factor which seemed to exert a protective role (Table 4).

## Discussion

The aim of the present study was to evaluate the prevalence of individuals at risk for EDs using the SCOFF screening questionnaire in a large, representative population of subjects aged 14–19. In addition, the study described this group in terms of associated interpersonal and psychological factors. In the analyzed sample, 19.4% of the boys and 44.6% of the girls had a SCOFF score above the cut-off, with greater percentages among higher BMI categories. Factors associated with this condition were experiencing bad family relationships, being teased by peers, having had the first sexual intercourse before the age of 14, and reporting high levels of perceived distress. Among young women, worse peer relationships were associated with a lower SCOFF score.

To the best of our knowledge, this was the first large epidemiological study to evaluate the prevalence of EDs symptoms in a representative sample of adolescents in Italy. These data confirmed that a remarkable percentage of individuals in this age range present significant body image and weight concerns, as seen in similar studies which, however, outlined a lower proportion of at-risk individuals [34–36]. It is possible to hypothesize that this alarming prevalence may be due to an improved awareness in the general population and to a reduced stigma in reporting ED symptoms, but it may also express a real increase in the occurrence of clinical and subthreshold ED symptoms [1, 8]. From this perspective, the rising incidence of EDs in adults may represent the tip of an iceberg with a greater number of vulnerable adolescents who are not detected—and, therefore, potentially not treated. It is noteworthy that—even though the present study confirmed a greater prevalence of ED symptoms among women—a remarkable percentage of boys expressed food- and body-centered concerns. This result is of particular interest and it cannot be neglected, given the fact that established gender-specific patterns, such as muscularity-oriented disordered eating [37], are not directly addressed by the SCOFF questionnaire. Moreover, this finding indirectly contributes to confirm that the gender gap in ED symptoms may be less pronounced in the general population as compared to clinical samples [38].

In both genders, SCOFF scores increased with BMI: this positive association can be interpreted considering that a large proportion of EDs may rise from overweight conditions and consequent diet attempts [9–11]. In fact, body dissatisfaction, weight concerns, and dietary restraints are clearly associated with weight status [39], potentially resulting in both restrictive [11] and binge-purge disordered eating [40]. In this sense, it is noteworthy that a higher BMI may be associated with worse ED-specific and general psychopathology in adolescents with AN, outlining the need for a close monitoring of atypical AN [41]. For this reason, the use of the questionnaire in this age group may be valuable for the early detection of ED symptomatology across the weight spectrum, i.e. irrespective of an established underweight condition due to dieting or strenuous physical activity.

In the present study, a higher parental education level was not associated with a condition of risk for EDs, a result which is not in line with a previous work on clinically established EDs [14]. In this sense, the cross-sectional design on a general population of adolescents may be inadequate to describe a complex and dynamic phenomenon which involves an expansion of education level in European countries and consequently reduced weight disparities [42]—obesity and overweight conditions being less represented among individuals with higher parental education [43]. For these reasons, extreme caution should be applied to the interpretation of this finding.

Moving forward in the characterization of high-risk conditions for EDs, it has been reported that adolescents often do not have a defined image or structured “behavior model”, and their cognitions and activities are, therefore, influenced by a larger number of environmental factors, as compared to adults [44]. Indeed, the present study confirmed that a perceived low quality of family relationships may be associated with altered eating and body weight attitudes [15–17]. Conversely, despite a different distribution among groups, worse peer relationships did not qualify as a risk factor for high SCOFF scores in the multivariable model, and bad relationships even proved to be protective for girls. The present finding is in contrast with a large body of evidence associating interpersonal difficulties—including peer relationships—with the onset [22] and the worsening [45] of ED symptoms. On the one hand, the self-reported categorical assessment of these dimensions does not allow a detailed comprehension of phenomena underlying the present findings. On the other hand, however, the data observed may be explained by the fact that this parameter can be strongly influenced by active choices of peer involvement (e.g., selection or avoidance), and it may, therefore, underlie coping strategies that cannot be applied in the domestic environment. The same holds true for victims of abusive behaviors from peers: indeed, bullying victimization was significantly associated with high SCOFF scores, confirming that weight and body image concerns cluster with this widespread dysfunctional interaction [20, 21]. Furthermore, the outlined association between EDs risk and a lower age at first sexual intercourse can be explained in the light of the interplay between early puberty and sexual risk-taking [23], which have been implicated in the onset of EDs psychopathology, often on the background of childhood traumatic experiences [24].

To conclude, ED symptomatology proved to be associated with high levels of psychological distress, as outlined through different study designs [2, 19, 46]. With this regard, the non-specific symptoms of anxiety and depression described by the K6 scale may indicate an underlying perceived distress: although at a speculative level, it is possible to hypothesize that these concerns may precede the development of more specific ED symptoms, such as over-preoccupation with body image and eating behavior, which are clearly represented among overweight adolescents who undergo dieting attempts [39]. In this sense, it is noteworthy that dietary restraint tied to high levels of psychological distress expresses a condition of risk for developing an ED [47], confirming the opportunity of a clinical evaluation for a part of subjects who undergo some form of dieting, and that preadolescent overweight and anxious distress recently proved to be associated with the development of ED symptoms in adolescence and young adulthood [48].

### Strength and limits

For the first time, the present study aimed to evaluate the prevalence of EDs symptoms in a large and representative sample of adolescents from the Italian general population, thus contributing to characterize these conditions which are increasingly emerging as a public health concern. In a constantly evolving epidemiological scenario, the study underlined that an alarming percentage of boys and girls expressed some body image concerns, even more than in other European studies [29, 30].

The present findings should be considered according to some limitations. First, the self-report of outcome measures may be associated with under-reporting due to perceived stigma tied to specific environmental factors (i.e. family and peer-relationships), pathological weight [49], or denial aimed at avoiding professional help for ED symptomatology. In this sense, the fact that some participants did not fill the SCOFF questionnaire may be an expression of selective non-response, even if no difference in this subsample was observed regarding randomization criteria. Second, the Italian version of the SCOFF questionnaire has low specificity (87.5%), and it may overestimate the number of individuals who deserve a clinical evaluation [30]. Third, the cross-sectional and observational study design does not allow to draw conclusions on the causative effect of the variables presented. To conclude, the broad categories used for the assessed variables may have produced a reduced variance, and a relative approximation of the investigated phenomena, as discussed for the evaluation of peer relationships. For this reason, multivariable analyses should be interpreted with caution.

### Conclusions

The present study detected a high prevalence of self-reported ED symptomatology among adolescents. This remarkable risk condition proved to be associated with an underlying pattern of perceived problematic family environment, bullying victimization, and higher levels of psychological distress. The identification of these factors may prove useful to ameliorate screening programs for the early detection of EDs, corroborating the need for a further clinical evaluation.

#### What is already known on this subject?

An increased incidence of EDs has been observed in the adolescent population during the last decades. In the past years, only a few studies used a validated screening questionnaire to produce an estimate of the prevalence of at-risk subjects in European countries, including Italy.

#### What this study adds?

This was the largest epidemiological study which provided the prevalence of subjects at risk for EDs in a representative population of adolescents of Italy. The study detected a remarkable and unexpectedly high prevalence of symptoms in both genders, and it provided a further characterization of putative risk factors among adolescents—thus underlining the need for a thorough screening activity and for future epidemiological monitoring.

## Data Availability

The data underlying this article will be shared on reasonable request to the corresponding author.

## Code availability

The code underlying this article will be shared at reasonable request to the corresponding author.
